# Triethoxysilyl-Functionalized Polyethylenimine: Its Spontaneous Cross-Linking and Drug Retention

**DOI:** 10.1021/acsomega.5c09741

**Published:** 2026-02-19

**Authors:** Erika Yoshihara, Ayaka Tomoda, Kana Morishita, Toshiyuki Takagi, Masayuki Sano, Kimio Sumaru

**Affiliations:** Cellular and Molecular Biotechnology Research Institute (CMB), National Institute of Advanced Industrial Science and Technology (AIST), Tsukuba Central 5, 1-1-1 Higashi, Tsukuba, Ibaraki 305-8565, Japan

## Abstract

Effective antibacterial and antiviral coatings are expected to serve as effective infection control measures in medical and public environments. In this study, we investigated polyethylenimine functionalized with triethoxysilyl groups (Si-PEI) as a promising platform material to implement such coatings feasibly. Si-PEI, synthesized in ethanol in a one-pot reaction, was coated onto the material surface as a diluted solution and then spontaneously cross-linked after solvent evaporation at room temperature. The resulting water-resistant coating layer retained water-soluble antimicrobial components such as copper­(II) ions and sulfonamide antibiotics within the cross-linked network, thereby moderately suppressing their leaching due to casual water exposure. Similar retention was also implemented for the antiviral agent didecyldimethylammonium chloride, and the coating layer demonstrated effective and stable suppression of viral infectivity of deposited droplets under realistic conditions.

## Graphical abstract

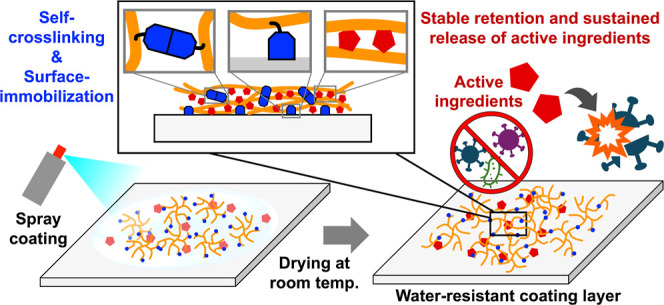

## Introduction

Various antimicrobial coating technologies have been developed to impart antimicrobial properties to material surfaces, with several already in commercial use. These coatings have been applied in medical contexts to prevent in vivo infections from grafts during surgical procedures^1^ and are actively being adapted for reducing nosocomial infections and improving hygiene standards in food processing environments.^2^ Furthermore, the global COVID-19 pandemic has drawn attention to coating technologies as an effective means of preventing contact transmission via surfaces such as doorknobs, and studies on their social implementation have been conducted.^3,4^ Even after the subsidence of COVID-19, there remains a need to develop new, more widespread, and high-performance technologies to prepare for future pandemics and address public health challenges in hospitals and nursing homes.

Meanwhile, the global emergence of antibiotic-resistant (AMR) bacteria poses a critical threat to healthcare systems. AMR is estimated to cause approximately 700,000 deaths annually worldwide, and projections suggest that this figure could rise to 10 million by 2050 in the absence of effective countermeasures.^5^ Given that current treatments for infectious diseases rely heavily on antibiotics, infections associated with AMR pose life-threatening risks, particularly for immunocompromised patients. Consequently, the development and implementation of strategies to mitigate contact-based transmission are becoming increasingly essential for interrupting nosocomial infection pathways.

Against this background, there is an increasing expectation for the development of technologies that can be applied in liquid form and remain stably immobilized on surfaces after drying to maintain their antimicrobial efficacy. Most antimicrobial coating technologies are based on polymeric approaches, with cationic polymers being the most widely employed. For instance, chitosan is a safe material obtained by deacetylation of crustacean chitin and has been considered for fruit and food coating applications.^6^ Similarly, polyarylamine, a synthetic polymer commonly used as an industrial raw material, has demonstrated high antimicrobial activity and stability when assembled into polyion complex multilayers via the layer-by-layer method.^7^

To facilitate broader societal adoption of these technologies, further enhancements in simplicity and versatility are essential. In this study, polyethylenimine (PEI) functionalized with triethoxysilyl groups (Si-PEI) was investigated as a polymer that is soluble in environmentally friendly solvents suitable for everyday use, applicable to object surfaces, and capable of spontaneous cross-linking under mild conditions, including room temperature.

PEI is a relatively inexpensive cationic polymer known for its potent antibacterial and antiviral properties.^8–11^ Meanwhile, triethoxysilyl groups possess the intrinsic ability to induce spontaneous cross-linking and to form stable bonds with glass and metal surfaces at ambient temperatures.^12,13^ Previous study has demonstrated that functionalizing PEI with triethoxysilyl groups enables its use as an additive to enhance the scratch resistance of epoxy resin coatings.^14^ In this method, 3-(triethoxysilyl)­propyl isocyanate was added to low molecular weight (800 g/mol) branched PEI at an equimolar ratio to its amino groups (corresponding to more than four times the weight of PEI) and allowed to react completely. As a result, a polymer material with a 100% modification ratio, rich in triethoxysilyl groups, was synthesized.

However, in this study aiming to realize antimicrobial coating technology, it is essential to consider not only the cross-linking capability but also the balance between stable retention and sustained release of active ingredients. Although it seems promising to employ PEI with a lower modification rate and higher molecular weight, it is challenging to uniformly introduce isocyanate, which is highly reactive with amino groups into a high viscosity, high molecular weight PEI solution. To address this issue, we explored an alternative synthetic route (Scheme 1) utilizing glycidyl and methacryl functional groups in place of isocyanate. The retention and sustained release behaviors of antimicrobial agents embedded in the resulting cross-linked polymer layers were evaluated using copper­(II) ions (Cu^2+^) among antimicrobial metal ions^15,16^ and representative antimicrobial compounds. In addition, the antiviral performance of Si-PEI cross-linked layers containing antiviral components was evaluated using a rapid and sensitive contact-infection assay involving recombinant Sendai virus (SeV) and virus-containing saliva model droplets.^17^

**Scheme 1:**
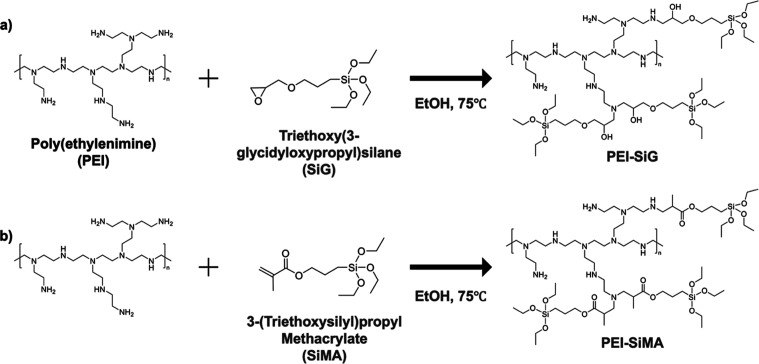
Triethoxysilyl-Functionalization of PEI: (a) PEI-SiG and (b) PEI-SiMA

## Materials and Methods

### Materials

Triethoxy­(3-glycidyloxypropyl)­silane (SiG), 3-(triethoxysilyl)­propyl methacrylate (stabilized with BHT) (SiMA), sulfasalazine (SASP), didodecyldimethylammonium chloride (DDDMA), and methanol-*d*_4_ 99.8 atom % D were purchased from Tokyo Chemical Industry Co., Ltd. (Tokyo, Japan). A branched PEI with average molecular weight ∼25,000 was purchased from Sigma-Aldrich (St. Louis, MO, USA). Another branched PEI with average molecular weight ∼10,000, ethanol (99.5%), and Dulbecco’s phosphate-buffered saline (D-PBS(−)) were purchased from Fujifilm Wako Pure Chemical Corporation (Osaka, Japan). Mucin, from Bovine Submaxillary Glands, was purchased from MP Bio Japan K.K. (Tokyo, Japan). Luminescent recombinant Sendai virus (Luc-SeV) was prepared according to previous literature.^17^ In order to measure the total protein concentration as an indicator of cell number, Pierce 660 nm Protein Assay Reagent (Thermo Fisher Scientific) was used.

### Methods

#### Preparation of Triethoxysilane-Functionalized PEI

Branched PEI with molecular weights of 10,000 and 25,000 was reacted with either SiG or SiMA to synthesize the corresponding polymers (PEI-SiG and PEI-SiMA). Each polymer was prepared via a simple one-pot addition reaction by mixing the specified ratios (Table 1) in ethanol, followed by heating at 75 °C for 2 h. The glycidyl group of SiG and the methacrylate group of SiMA underwent cycloaddition and Michael addition reactions, respectively, with the primary amine groups of PEI to form PEI-SiG and PEI-SiMA.^18,19^ The incorporation of triethoxysilyl groups into PEI was confirmed from ^1^H NMR spectra of the reaction mixture in methanol-*d*_4_ measured by using a ^1^H NMR spectrometer (AVANCE III HD 600; Bruker, Billerica, Massachusetts, USA). Successful modification was indicated by the disappearance of characteristic proton signals corresponding to the epoxy group (3.14 ppm) and the methacryl group (5.63 and 6.11 ppm), as shown in Figures S1 and S2. In addition, a PEI-SiG solution was coated onto the surface of a polystyrene dish, and the effect of drying conditions on the water and ethanol resistance of the resulting coated layer was investigated.

**Table 1:** Feed Ratio of Triethoxysilane-Modified PEI

sample	molecular weight of PEI (kDa)	SiG/SiMA functionalization (mol %)	averaged number of SiG/SiMA per molecule of PEI
PEI-SiG10k20	10	20	11
PEI-SiG25k30	25	30	19
PEI-SiG25k40	25	40	14
PEI-SiMA10k10	10	10	23

#### Evaluation of Polymer Elution from PEI-SiG-Coated Layer

The stabilization of the coating layer, as spontaneous cross-linking of PEI-SiG progressed, was quantitatively evaluated from the proportion of polymer eluted by water washing. PEI derivatives exhibit a strong chelating affinity for Cu^2+^ ions, and the resulting complexes display UV–vis absorbance spectra distinct from those of free Cu^2+^. Leveraging the pronounced absorbance peak at 270 nm, the concentration of PEI-SiG eluted from cross-linked coating layers under various conditions was quantitatively determined. UV–vis absorbance spectra were measured for aqueous solutions containing PEI-SiG at multiple concentrations, in the presence of a fixed concentration (0.32 mM) of copper­(II) sulfate (CuSO_4_), using a spectrometer (V-750, JASCO Corporation, Tokyo, Japan) and a cuvette with a 10 mm optical path length. The absorbance values at 270 and 480 nm (denoted as *A*_270_ and *A*_480_, respectively) were correlated with the PEI-SiG concentration (*C*_p_) according to the following relationship

$$C_{p}\;\left(wt\;\%\right)=0.0062\left(A_{270}-A_{480}-0.06\right),where\;C_{p}<0.014\;wt\;\%$$

A 2% (w/v) ethanol solution of PEI-SiG10k20 (7.4 mg) was drop-cast onto a 35 mm diameter polystyrene dish (surface area: 10 cm^2^), followed by spin-coating at 800 rpm. The coated dishes were then left to stand at room temperature (25 °C) for varying durations of 0, 1, 2, and 19 h to facilitate cross-linking of the coating layer (*N* = 3 for each condition). After incubation, water was added, and the eluted PEI-SiG was recovered by rinsing the surface. The eluate was mixed with a CuSO_4_ solution and diluted to a final Cu^2+^ concentration of 0.32 mM in a total volume of 3 mL. UV–vis absorbance spectra of the resulting solution were measured, and the concentration of eluted PEI-SiG was calculated using the previously established formula to estimate the coating layer’s elution rate.

Additionally, to evaluate the stability of polymer coating layers cross-linked under different environmental conditions, PEI-SiG10k20, PEI-SiG25k30, and PEI-SiG25k40 were coated onto substrates under the same conditions described as above. The surfaces were then left for 5 min, 30 min, and 12 h at 20 °C and for 1 h at 45 °C to promote the cross-linking reaction of the coating layers. Furthermore, the elution rate of the coating layers was measured using the same method described above to estimate the percentage of the residual polymer. The solution coating was confirmed to be applied uniformly across the substrate surface. Therefore, all of the solute components were considered to be present in the coating layer. Based on the solute’s areal density, the average film thickness was estimated to be approximately 0.14 μm. In addition, the coating layer was observed to remain transparent and homogeneous both before and after cross-linking.

#### Evaluation of Cross-Linked PEI-SiG and PEI-SiMA Layers Surface Using Contact Angle Measurement

A 2% (w/v) ethanol solution of various triethoxysilane-functionalized PEI (7.4 mg) was drop-cast onto a 35 mm diameter polystyrene dish (surface area: 10 cm^2^), followed by spin-coating at 800 rpm. The coated dishes were then incubated at 80 °C for 1 h to facilitate cross-linking of the coating layer, thereby preparing water-resistant PEI-SiG and PEI-SiMA surfaces.

For contact angle analysis, 5 μL droplets of water were deposited onto the samples by using a contact angle meter (DropMaster 300, Kyowa Interface Science Co., Ltd., Saitama, Japan). The resulting images of the liquid–air interface were analyzed with FAMAS software. Contact angles were measured in three different areas of each sample, and the values were averaged. As shown in Figure S3, the contact angles of all surfaces coated with triethoxysilane-functionalized PEI were reduced compared to noncoated polystyrene surface corresponding to the immobilization of PEI, which is a water-soluble polymer. Furthermore, the sample prepared with PEI 25,000 and smaller amount of triethoxysilyl group exhibited lower contact angles than that with PEI 10,000 and larger amount of triethoxysilyl group, indicating that longer PEI chains between cross-linking points confer greater hydrophilicity.

#### Evaluation of Copper Ion Capture from Aqueous Solution by Cross-Linked PEI-SiG Layers

A 10 μL aliquot of a 5 wt % ethanol solution of PEI-SiG25k30 was loaded onto the center of a 35 mm diameter polystyrene dish (bottom area: 10 cm^2^) and heated on a hot plate at 40 °C for 2 h to form a uniform coating layer approximately 30 mm in diameter. The resulting layer exhibited complete resistance to both water and ethanol. To evaluate Cu^2+^ adsorption properties based on its known antimicrobial activity, 1.5 mL of a 0.5 mM CuSO_4_ solution was added to the coated surface. As a control, an uncoated dish was subjected to the same procedure. After stirring for 20 min to ensure full contact, the solution was collected and mixed with 1.5 mL of water containing 0.4 mg of PEI (average molecular weight: 10 kDa), an amount sufficient to chelate Cu^2+^ and induce visible darkening of the solution. The mixture was subjected to UV–vis spectrophotometric analysis, and the residual Cu^2+^ concentration was estimated from the absorbance at 270 nm, which corresponds to Cu^2+^ chelated by unpaired amino electron pairs.

#### PEI Evaluation of SASP Retention by Cross-Linked PEI-SiG Layers

Ethanol-based coating solutions were prepared containing 5 wt % PEI-SiG10k20 and 1 wt % salazosulfapyridine (SASP). A 10 μL aliquot of each solution was loaded onto the center of a 35 mm diameter polystyrene dish and spin-coated at 800 rpm under a nitrogen atmosphere. For comparison, a solution without PEI-SiG10k20 was prepared by adding 1.5 volumes of 1 N sodium hydroxide relative to SASP. Ethanol was subsequently added to adjust the SASP concentration to 1 wt %. A 10 μL aliquot of the resulting solution was coated onto a polystyrene dish under the same conditions as described above. Each coated dish was subsequently placed on a hot plate at 40 °C and incubated for 12 h to allow complete drying and cross-linking. To evaluate the elution behavior of SASP, 1.5 mL of water was added to each coated surface and stirred. The eluate was collected, followed by rinsing with an additional 1.5 mL of water, yielding a total volume of 3 mL per sample. To facilitate complete dissociation of SASP’s acidic functional groups, 50 μL of 0.01 N sodium hydroxide was added to each solution. The resulting aqueous samples were analyzed by UV–vis spectrophotometry, and the SASP elution rates were estimated based on absorbance values at 365 nm.

#### Evaluation of Antiviral Properties of DDDMA-Retaining Cross-Linked PEI-SiMA Layers

Ethanol solutions were prepared containing the following compositions: (1) 0.1 wt % DDDMA, (2) 0.1 wt % DDDMA plus 0.45 wt % polyethylenimine (PEI; average molecular weight: 1000), (3) 0.45 wt % PEI-SiMA10k10, and (4) 0.45 wt % PEI-SiMA10k50. A 4 μL aliquot of each solution was applied to six wells in a 48-well plate and left to stand at room temperature for half a day. Subsequently, to simulate situations where exposure to water occurs, such as by touching with wet hands, 1 mL of water was added to three of the six wells for each solution condition, shaken for 10 s, and then removed to form a coating layer. The antiviral effect of these layers was evaluated according to the method described in a previous report.^17^ Specifically, a 4:1 mixture of a 0.25 wt % Dulbecco’s buffered solution of bovine submaxillary gland mucin and Luc-SeV-containing medium was prepared. This mixture, previously confirmed to exhibit virus-protective properties, served as a model saliva droplet solution containing an enveloped virus. A 2 μL aliquot of the mixture was then dispensed onto the previously prepared coating layer. After 1 h of incubation with the lid on, 10 μL of Dulbecco’s buffer solution was added to each well and collected. The resulting sample was introduced into the medium of the cells cultured in 96-well plates. On the following day, luciferase luminescent activity was measured using a multiwell microplate reader (Synergy H1; BioTek, Winooski, VT, USA) and normalized to the number of cells, serving as an index of residual viral activity. Pierce 660 nm Protein Assay was performed to measure the protein concentration as an indicator of cell number.

## Results and Discussion

### Evaluation of Polymer Cross-Linking Progress of PEI-SiG

The progression of stabilization of cross-linking in PEI-SiG-coated layers was assessed by quantifying the percentage of polymer eluted after water rinsing, as shown in Figure 1. Although the PEI-SiG coating layer was almost completely dissolved in water immediately after coating, significant cross-linking occurred after standing for 1 h at 25 °C, resulting in approximately 80% of the PEI-SiG remaining on the polystyrene substrate surface following rinsing. This observation indicates that cross-linking between polymers via triethoxysilyl groups proceeds on a time scale of 1 h at room temperature, even after solvent evaporation. It was also observed that the polymer was hardly eluted by water rinsing after 19 h, suggesting that the coated PEI-SiG contained few molecules lacking triethoxysilyl groups. This observation aligns with the synthesis conditions, in which triethoxysilyl groups were introduced at a ratio of one per 20 monomer units in PEI with a weight-average molecular weight of 20 kDa (∼470 monomer units). In addition, Figure 2 summarizes the effects of PEI molecular weight, triethoxysilyl group substitution ratio, incubation time, and temperature on the stability of the resulting cross-linked coating layers. Initially, all PEI-SiG coatings rapidly dissolved in water upon solvent evaporation. However, over time, spontaneous cross-linking significantly improved the coatings’ resistance to both water and ethanol. The water and ethanol resistance of the formed PEI-SiG coating layer increased with the molecular weight of the polyethylenimine as the raw material and with a higher modification ratio by SiG. In particular, coatings prepared under conditions of PEI-SiG10k20, PEI-SiG25k30, and PEI-SiG25k40 demonstrated complete water and ethanol resistance when incubated at 45 °C for 1 h or at 20 °C for 12 h. These results indicate that the ethanol solution of PEI-SiG can form a stable coating layer via self-cross-linking under mild temperature conditions of approximately room temperature after coating onto the substrate surface. In all conditions, the average number of SiG or SiMA units introduced per PEI molecule exceeded 10 (see Table 1). Even considering the broad molecular weight distribution of PEI, this suggests that nearly all polymer molecules participated in cross-linking, supported by the experimental result.

**Figure 1:**
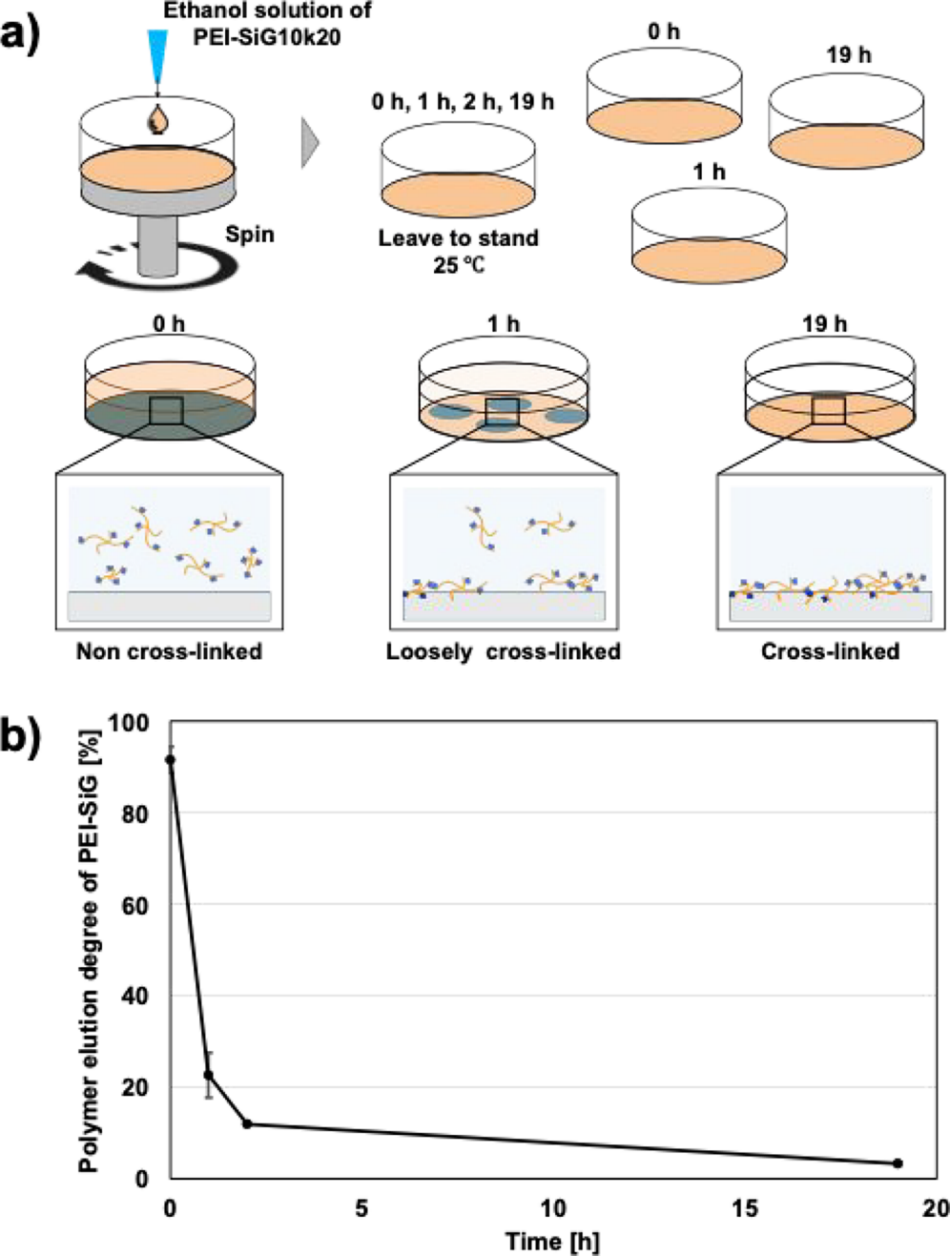
(a) Schematic illustration of this measurement. (b) Influence of cross-linking time on polymer elution rate from PEI-SiG10k20-coated layers upon flushing with water (25 °C, mean ± SD, *n* = 3).

**Figure 2:**
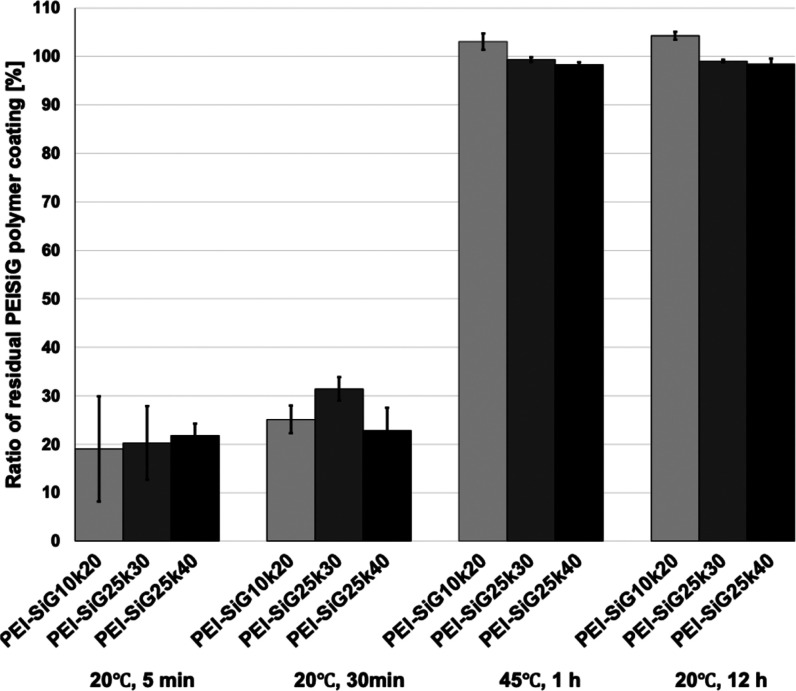
Water resistance of cross-linked PEI-SiG coating layers with different SiG compositions (mean ± SD, *n* = 3).

With respect to the stability of the coating layer, polymer cross-linking and bonding to the substrate are separate phenomena. On the other hand, as seen in photoresist development, it is widely known that when polymers cross-link within a coating layer, they adhere to the object surface even without a chemical bond and become difficult to remove by flushing with a good solvent. The triethoxysilyl group used as the cross-linking motif in this study forms stable bonds with glass and metal surfaces, but polystyrene was used as the model substrate in this study. Therefore, it is presumed that the stabilization is primarily due to the effect of polymer cross-linking alone.

### Copper Ion Collection Characteristics from Solution by Cross-Linked PEI-SiG Layers

Given the strong chelating affinity of PEI derivatives for Cu^2+^, a metal ion known for its antimicrobial efficacy, the cross-linked PEI-SiG coating layer is expected to exhibit antimicrobial activity through stable Cu^2+^ retention. To evaluate the Cu^2+^ retention capacity of the cross-linked PEI-SiG layer, the amount of Cu^2+^ adsorbed from a 0.5 mM CuSO_4_ solution applied to the material surface was quantified. Figure 3 shows the results of the determination of CuSO_4_ concentration in the aqueous solution collected after stirring for 20 min on the polystyrene surface with and without the cross-linked PEI-SiG layer, respectively. In the absence of the cross-linked PEI-SiG layer, all Cu^2+^ present in the original CuSO_4_ solution was recovered (100% remaining in the aqueous phase), confirming that the polystyrene surface does not adsorb Cu^2+^ ions. In contrast, the cross-linked PEI-SiG layer adsorbed over 90% of Cu^2+^ from the aqueous solution, leaving approximately 8% remaining, and was colored blue by chelate-stabilized Cu^2+^. In addition, Cu^2+^ extraction was attempted by loading 1.5 mL of water onto the dish with Cu^2+^ adsorbed on the cross-linked PEI-SiG layer supported on the bottom and stirring for 5 min. As a result, only approximately 5% of the total amount adsorbed could be extracted, indicating that most of Cu^2+^, which is an antimicrobial component in water-soluble form, is stably retained in the cross-linked PEI-SiG layer. Water-soluble polymers are known to form complexes with various metal ions, and PEI exhibits a strong chelating affinity for Cu^2+^, with reported binding constants exceeding 10^20^ mol/L. From this, it is suggested that cross-linked PEI-SiG layers strongly stabilize Cu^2+^ ions.

**Figure 3:**
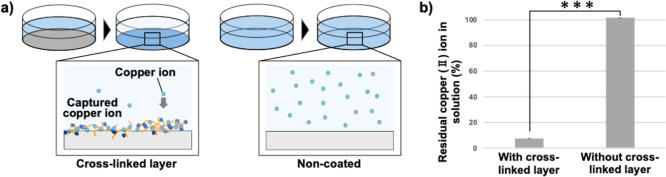
(a) Schematic illustration of this measurement. (b) Measurement of copper ion capture from aqueous solution (mean ± SD, *n* = 3, ***p < 0.001).

### Inhibition of Elution of Antimicrobial SASP by Cross-Linked PEI-SiG Layer

To evaluate the retention characteristics of cross-linked PEI-SiG layers with respect to the sulfonamide antimicrobial agent SASP, the concentration of SASP eluted from air-dried surfaces coated with a mixture of PEI-SiG and SASP or with SASP alone is shown quantitatively in Figure 4. Since SASP is a water-soluble small molecule, all SASP coated to the surface eluted from the surface into water under the SASP-only coating condition. In contrast, when five times the amount of PEI-SiG was coated, only approximately 1% of SASP eluted. The following retention mechanism has been proposed to explain the stable surface retention of highly water-soluble SASP. Proton transfer occurs from SASP, a proton donor, to PEI, a proton acceptor, resulting in the formation of SASP anions and PEI cations. The electrostatic interaction between these oppositely charged species enables SASP to be stably retained within the mesh structure of the cross-linked PEI-SiG layer, even upon contact with water. Based on these results, coating with a mixture of PEI-SiG has been demonstrated to be a simple and effective method for stably retaining water-soluble, low-molecular-weight substances on object surfaces.

**Figure 4:**
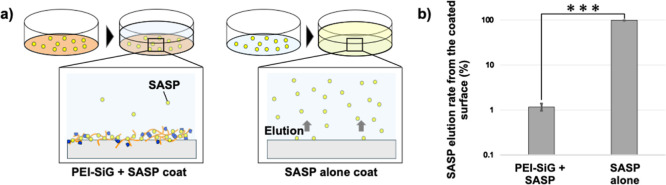
(a) Schematic illustration of this measurement. (b) Measurement of SASP elution rate from the coated surface (mean ± SD, *n* = 3, ***p < 0.001).

### Maintenance of Antiviral Properties by Retention of DDDMA

To evaluate the retention properties of the antiviral agent DDDMA, a virus infection test using Luc-SeV was conducted on a coating layer of PEI-SiMA. As described above, a model solution mimicking the saliva droplet solution containing Luc-SeV and bovine submaxillary gland mucin, which has a high viral protection effect, was dropped onto the PEI-SiMA-coated layer. It was allowed to stand for 1 h, after which the solution was collected and added to cultured cells, and the luciferase luminescence activity was measured as an indicator of residual viral activity. The results are shown in Figure 5. DDDMA alone exhibited high antiviral activity, reducing residual viral infectivity to below the detection limit (∼1 × 10^2^). However, after flushing to simulate situations where exposure to water occurs, such as by touching with wet hands, the surface’s antiviral activity was only about one-fifth that of the uncoated condition, suggesting that a significant portion of the DDDMA coating was lost during flushing. In addition, slight residual infectivity was observed in the mixture containing nonbridging PEI. Furthermore, under nonflush conditions, the strong cytotoxicity of PEI introduced together with the virus led to a reduction in the number of infected cells to approximately 60% compared to other conditions (Figure S4), suggesting that compromised cell membrane integrity may have influenced the estimation of residual infectivity. On the other hand, in the mixture with PEI-SiMA, residual viral infectivity was reduced below the detection limit, regardless of flushing. This result indicates that DDDMA trapped within the PEI-SiMA coating layer is not readily flushed but instead is gradually released into adhered droplets, allowing it to reach and inactivate viruses shielded by mucin.

**Figure 5:**
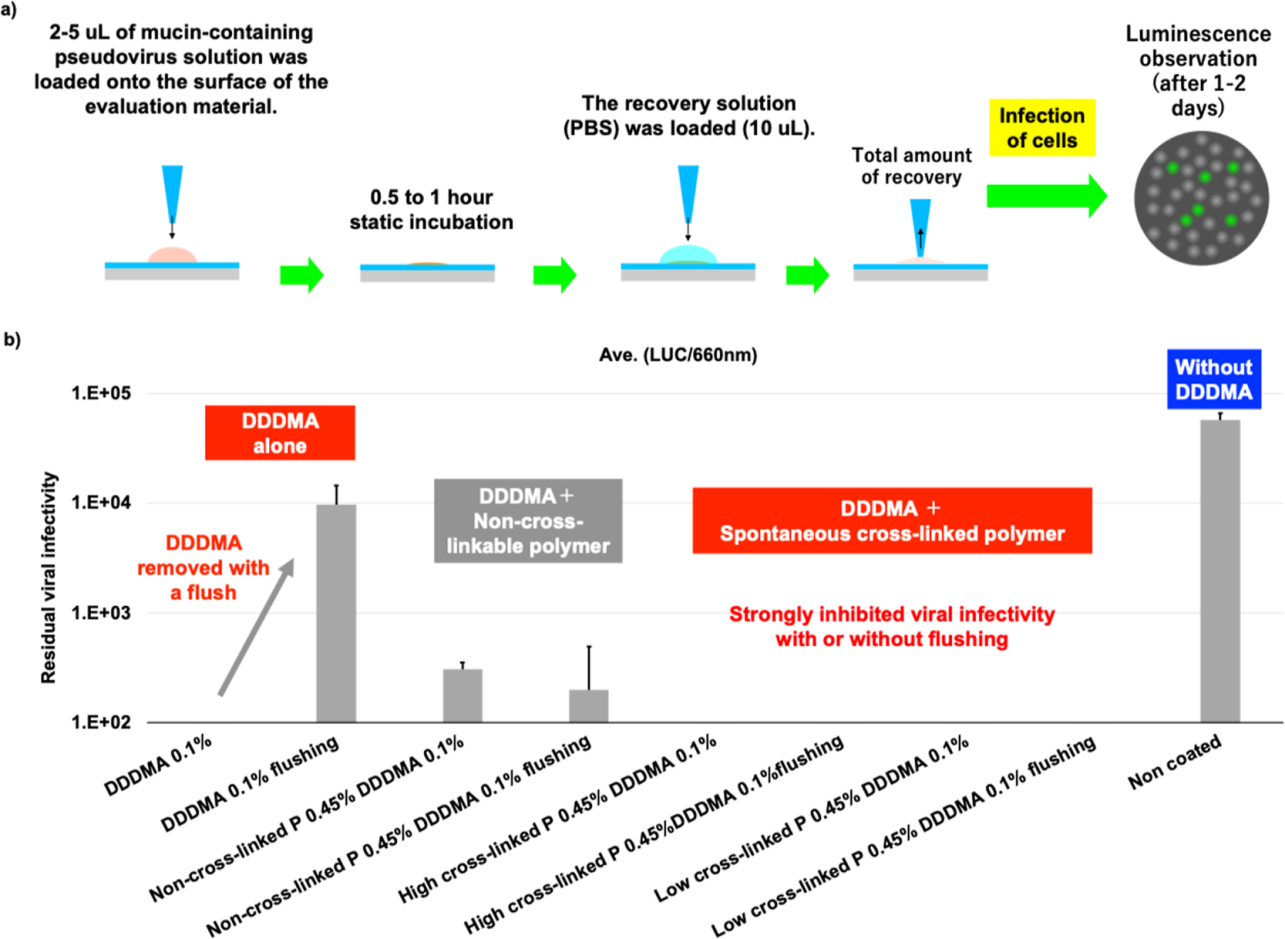
(a) Schematic illustration of novel assay method using luminescent pseudoviruses. (b) Luciferase luminescence activity under various conditions (mean ± SD, *n* = 3). Comparisons were made with and without flushing.

## Conclusions

This study demonstrated the development and functional evaluation of an antibacterial and antiviral surface coating system based on polyethylenimine functionalized with triethoxysilyl groups, Si-PEI. When applied as an ethanol solution, Si-PEI underwent spontaneous cross-linking under mild conditions (∼room temperature), forming stable coating layers resistant to water and ethanol exposure. These cross-linked Si-PEI layers effectively retained water-soluble antimicrobial agents, including copper­(II) ions and sulfonamide compounds. Furthermore, a cross-linked Si-PEI layer coated with DDDMA was shown to reduce the infectivity of viruses in saliva-like adherent droplets through the stable retention and gradual release of DDDMA. Sendai virus, like COVID-19, is an enveloped RNA virus and is expected to be similarly affected by physicochemical inactivation methods such as the technique examined in this study. Sufficient effectiveness in suppressing infection via these pathways for enveloped viruses that may pose future threats can be anticipated. These results strongly suggest that Si-PEI is a promising new material capable of forming a cross-linked layer that stably retains various antibacterial and antiviral components simply through the application of a mixed solution. This technology is expected to be deployed as an infection control strategy in public health settings, including hospitals and nursing care facilities.

## Supplementary Material



## Notes

^1^H NMR spectrum of samples, contact angle images for droplets water, and result of protein assay for the cells (PDF)

## References

[1] Hetrick E. M., Schoenfisch M. H. (2006). Reducing Implant-Related Infections: Active Release
Strategies. Chem. Soc. Rev..

[2] Page K., Wilson M., Parkin I. P. (2009). Antimicrobial Surfaces and Their
Potential in Reducing the Role of the Inanimate Environment in the
Incidence of Hospital-Acquired Infections. J.
Mater. Chem..

[3] Behzadinasab S., Chin A., Hosseini M., Poon L., Ducker W. A. (2020). A Surface
Coating That Rapidly Inactivates SARS-CoV-2. ACS Appl. Mater. Interfaces.

[4] Elsevier
BV (2020). Anti-Viral Surface Coating
to Prevent the Spread of COVID-19. Focus Powder
Coating..

[5] Rakowska P. D. (2021). Antiviral Surfaces and
Coatings and Their Mechanisms of Action. Commun.
Mater..

[6] Devlieghere F., Vermeulen A., Debevere J. (2004). Chitosan: Antimicrobial Activity,
Interactions with Food Components and Applicability as a Coating on
Fruit and Vegetables. Food Microbiol..

[7] Lichter J. A., Van Vliet K. J., Rubner M. F. (2009). Design of Antibacterial Surfaces
and Interfaces: Polyelectrolyte Multilayers as a Multifunctional Platform. Macromolecules.

[8] Gibney K. A. (2012). Poly­(ethylene imine)­s as Antimicrobial Agents with Selective Activity. Macromol. Biosci..

[9] Müller M., Urban B., Mannala G. K., Alt V. (2021). Poly (ethyleneimine)/poly
(acrylic acid) multilayer coatings with peripherally bound staphylococcus
aureus bacteriophages have antibacterial properties. ACS Appl. Polym. Mater..

[10] Mouritz A. P. (2021). Towards Antiviral Polymer Composites to Combat COVID-19 Transmission. Nano Sel..

[11] Pranantyo D., Zhang K., Si Z., Hou Z., Chan-Park M. B. (2022). Smart multifunctional
polymer systems as alternatives or supplements of antibiotics to overcome
bacterial resistance. Biomacromolecules.

[12] Sypabekova M., Hagemann A., Rho D., Kim S. (2023). 3-Aminopropyltriethoxysilane
(APTES) deposition methods on oxide surfaces in solution and vapor
phases for biosensing applications. Biosensors.

[13] Wang W., Vaughn M. W. (2008). Morphology and Amine Accessibility
of (3-Aminopropyl)
Triethoxysilane Films on Glass Surfaces. Scanning.

[14] Acebo C. (2014). Novel Epoxy-Silica Hybrid
Coatings by Using Ethoxysilyl-Modified
Hyperbranched Poly­(ethyleneimine) with Improved Scratch Resistance. Polymer.

[15] Li N., Pranantyo D., Kang E. T., Wright D. S., Luo H. K. (2020). A simple
drop-and-dry approach to grass-like multifunctional nanocoating on
flexible cotton fabrics using in situ-generated coating solution comprising
titanium-oxo clusters and silver nanoparticles. ACS Appl. Mater. Interfaces.

[16] Ermini M. L., Voliani V. (2021). Antimicrobial nano-agents: the copper age. ACS Nano.

[17] Sano M. (2022). Rapid and Highly Sensitive Method for Evaluating Surface Coatings
Against an Enveloped RNA Virus. ACS Appl. Bio
Mater..

[18] Du Y. (2023). Ring-Opening
Mechanism of Epoxides with Alcohol and Tertiary Amines. Polym. Chem..

[19] Fedotova A. (2018). Solvent Effects in the Aza-Michael Addition of Anilines. C. R. Chim..

