# Supplementary material for: Triethoxysilyl-Functionalized Polyethylenimine: Its Spontaneous Cross-Linking and Drug Retention
Source: ACS Omega. 2026 Feb 19;11(8):13268–74. doi: 10.1021/acsomega.5c09741 (PMC12961542; doi:10.1021/acsomega.5c09741)
Supplement: Supplementary file 1 [file ao5c09741_si_001.pdf]

Supporting Information

Triethoxysilyl-functionalized polyethyleneimine ：its spontaneous crosslinking and drug retention

*Erika Yoshihara, Ayaka Tomoda, Kana Morishita, Toshiyuki Takagi, Masayuki Sano and Kimio Sumaru**

Cellular and Molecular Biotechnology Research Institute (CMB), National Institute of Advanced Industrial Science and Technology (AIST), Tsukuba Central5, 1-1-1 Higashi, Tsukuba, Ibaraki 305-8565, Japan

*Kimio SUMARU, Tel:+ 81-80-2201-9816, E-mail: k.sumaru@aist.go.jp

Table of contents:

Figure S1. ^{1}H-NMR spectrum of a) SiG and b) PEI-SiG in Methanol-d_4.

Figure S2. ^{1}H-NMR spectrum of a) SiMA and b) PEI-SiMA in Methanol-d_4.

Figure S3. Contact angle images for droplets water: non coated dish surface, crosslinked PEI-SiG PEI-SiG, and crosslinked PEI-SiMA coating dish surface (mean± SD, n = 3).

Figure S4. The absorbance at 660 nm in Pierce 660 nm Protein Assay as an indicator of cell number (mean ± SD, n = 3). Comparisons were made with and without flushing.

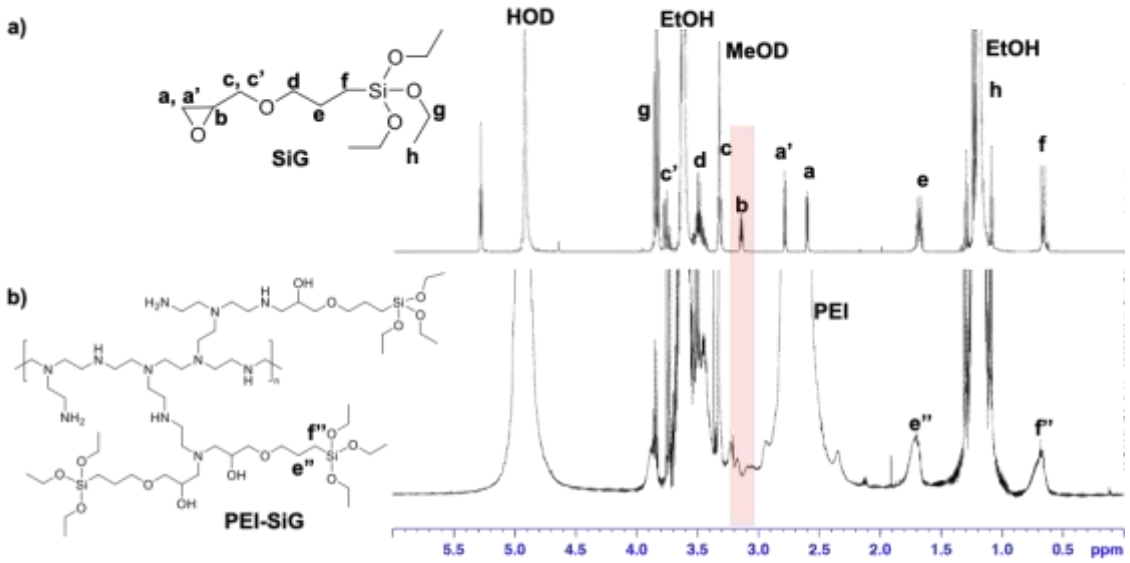

Figure S1. ^{1}H-NMR spectrum of a) SiG and b) PEI-SiG in Methanol-d_4.

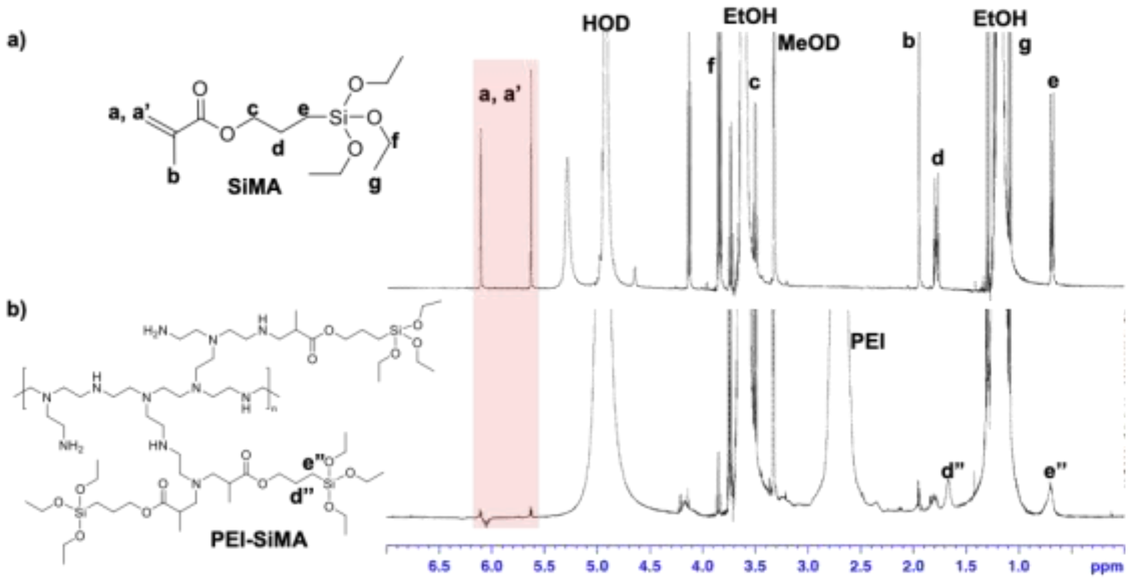

Figure S2. ^{1}H-NMR spectrum of a) SiMA and b) PEI-SiMA in Methanol-d_4.

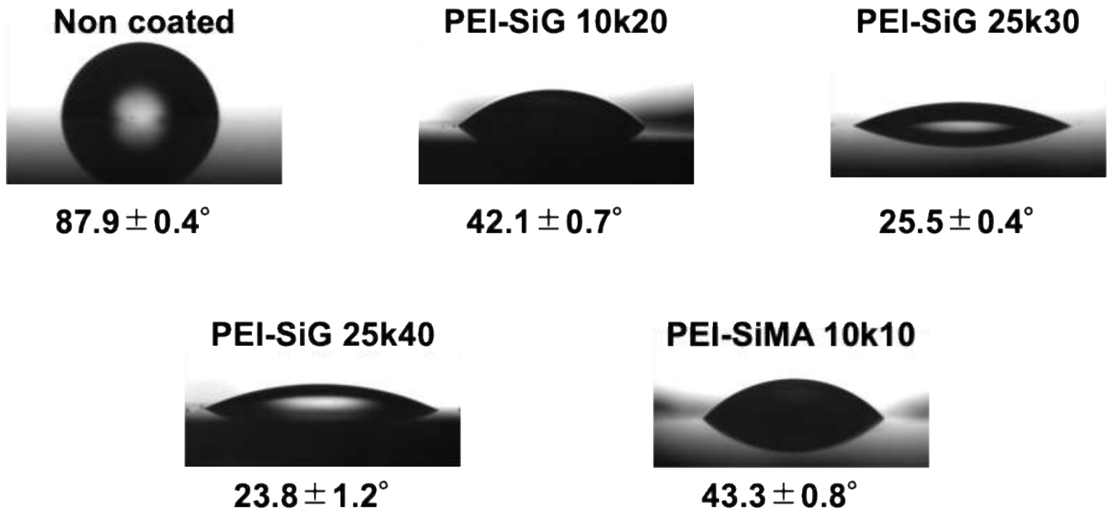

Figure S3. Contact angle images for droplets water: non coated dish surface, crosslinked PEI-SiG, and crosslinked PEI-SiMA coating dish surface (mean± SD, n = 3).

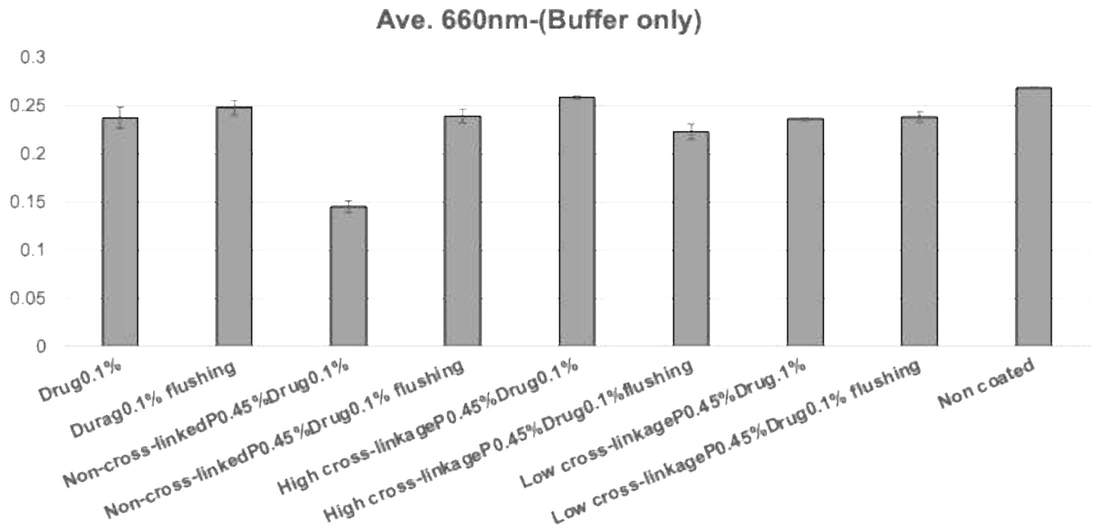

Figure S4. The absorbance at 660 nm in Pierce 660 nm Protein Assay as an indicator of cell number (mean± SD, n = 3). Comparisons were made with and without flushing.
